# Polymer-Based Scaffolds Loaded with *Aloe vera* Extract for the Treatment of Wounds

**DOI:** 10.3390/pharmaceutics13070961

**Published:** 2021-06-26

**Authors:** Sibusiso Alven, Vuyolwethu Khwaza, Opeoluwa O. Oyedeji, Blessing A. Aderibigbe

**Affiliations:** Department of Chemistry, University of Fort Hare, Alice 5700, Eastern Cape, South Africa; 201214199@ufh.ac.za (S.A.); vkhwaza@ufh.ac.za (V.K.); ooyedeji@ufh.ac.za (O.O.O.)

**Keywords:** *Aloe vera*, wound treatment, wound dressings, nanofibers, hydrogels, films, polymers

## Abstract

The treatment of wounds is one challenging biomedical field due to delayed wound healing common in chronic wounds. Several factors delay wound healing, including microbial infections, malnutrition, underlying physiological conditions, etc. Most of the currently used wound dressing materials suffer from poor antimicrobial properties, poor biodegradability and biocompatibility, and weak mechanical performance. Plant extracts, such as *Aloe vera,* have attracted significant attention in wound management because of their interesting biological properties. *Aloe vera* is composed of essential constituents beneficial for the wound healing process, such as amino acids, vitamins C and E, and zinc. *Aloe vera* influences numerous factors that are involved in wound healing and stimulates accelerated healing. This review reports the therapeutic outcomes of aloe vera extract-loaded polymer-based scaffolds in wound management.

## 1. Introduction

The skin is exposed constantly to injuries that result in a disruption in the epidermal and dermal lining of the skin [1]. If the connective and epithelium and connective structures are impaired, the defense offered to the human body from the surrounding environment becomes weakened. Wounds are typically categorized based on their nature and healing period as acute or chronic wounds [2]. Acute and chronic injuries need good clinical management that mainly involves the use of wound dressings. About 5.7 million people in the USA are affected by chronic wounds, and approximately 20 billion dollars is spent annually on wound management [3]. Ideal wound dressings should display beneficial properties for an effective wound healing process, especially for chronic wounds. The properties of ideal wound dressing materials include good antimicrobial effects, moderate water vapor transmission rate, good mechanical properties, high porosity, high water-absorbing capacity, allow gaseous exchange, ability to offer a moist environment, non-immunogenicity, good biodegradability and biocompatibility, and non-toxicity [4,5,6,7,8].

Wound dressings that display the properties mentioned above are commonly prepared from natural and synthetic polymers [9]. The natural polymers used in the fabrication of wound dressing scaffolds include hyaluronic acid (HA), chitosan, cellulose, fibrin, gelatin, dextran, elastin, etc. [10]. The dressings that are prepared from these polymers suffer from poor mechanical properties that are easily overcome by combining them with synthetic polymers, including plasticizers (e.g., polyethylene glycol or glycerol) [11]. The synthetic polymers that can be utilized in wound dressings are poly(lactic-*co*-glycolic acid) (PLGA), poly(ethylene oxide) (PEO), polyglycolic acid (PGA), polylactide (PLA), poly(hydroxyethyl methacrylate) (pHEMA), poly(ε-caprolactone), (PCL), poly(vinyl alcohol) (PVA), etc. [12]. The poor biological activities of polymer-based wound dressing materials can be overcome by incorporating bioactive agents in these materials. Plant extracts are bioactive agents that are frequently loaded in wound treatment because of their interesting therapeutic properties.

*Aloe vera* (AV) is a plant extract that is used in biomedical applications such as anticancer, antimicrobial antiviral as well as wound healing due to its numerous components [13]. The biological activities of AV include analgesic, antibacterial, antioxidant, antifungal, antiviral, wound healing, anti-inflammatory, cleansing, antiseptic, etc. [14]. AV affects various factors that are involved in the wound healing process and promotes healing. There are various classes of polymeric wound dressing materials that can be enriched with this plant extract to enhance their wound healing properties, such as hydrogels, nanofibers, films, membranes, foams, wafers, and transdermal patches [4]. This review is focused on the in vitro and in vivo therapeutic outcomes of polymer-based wound dressings encapsulated with AV.

## 2. Phases of Wound Healing Process

The duration of wound healing depends on whether the injury is an acute or chronic wound. An acute wound heals at an expected timeframe of 2 to 3 months and is caused by trauma, such as corrosive chemicals, surgery, stabbing, burns, etc. [15]. Acute wounds can become chronic wounds due to various factors, such as underlying physiological diseases, malnutrition, microbial infections, obesity, aging, and smoking.

**Figure 1:**
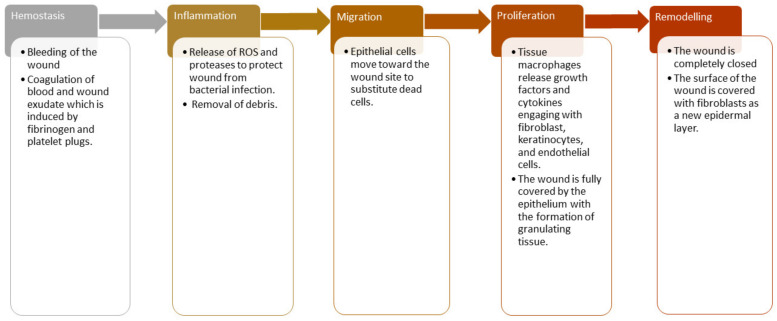
Phases of wound healing.

The healing process of chronic wounds takes more than three months. Some examples include diabetic ulcers, decubitus ulcers, and arterial ulcers, significantly reducing patients’ life quality [16]. The wound-healing process is a complex cascade of cellular events composed of reconstitution, resurfacing, and the restoration of the normal form of damaged skin [17]. The wound-healing process is divided into five sequential phases that can also overlap: hemostasis, inflammation, proliferation, migration, and remodeling phase (Figure 1) [17,18].

The hemostasis phase happens instantly after the injury, and it usually takes place at the same time as the inflammation phase. A major constituent of the skin connective tissue known as fibrinogen and platelet plugs promotes coagulation of wound exudate and blood to close damaged blood vessels and terminate bleeding [19]. The process of vasoconstriction occurs during this hemostasis phase. The hemostasis phase usually occurs for two days or more, depending on the gravity of the lesion. The next phase of the wound healing process is the inflammation phase, the phagocytic cells release reactive oxygen species and proteases to cleanse off the debris and protect the wound from microbial infections [20]. This phase is characterized by the swelling, warmth, and redness of the injured site caused by wound exudate (white blood cells). The macrophages that are differentiated from monocyte cells release growth factors and cytokines, recruiting endothelial cells, fibroblasts, and keratinocytes to restore disrupted blood vessels [21].

The third phase of wound-healing is the migration phase, and the epithelial cells move towards the injured site. The migration phase is followed by the proliferation phase [22]. In the proliferation stage, the destroyed blood vessels regenerate when the tissue macrophages transform from white blood cells, release growth factors and cytokines, engage with fibroblast, keratinocytes, and endothelial cells. In this phase, the lesion is fully covered by the epithelium with the development of granulating tissue and new tissue formation. The freshly developed tissue usually is pink or red when it covers the wound site [23]. The last stage of wound healing is the maturation phase that is also known as the remodeling phase. In this phase, the wound is completely closed, and the surface of the wound is covered with fibroblasts as a new epidermal layer of the skin. There are different wound dressings suitable for each phase of the wound healing process [24].

## 3. Classification of Wound Dressings

**Figure 2:**
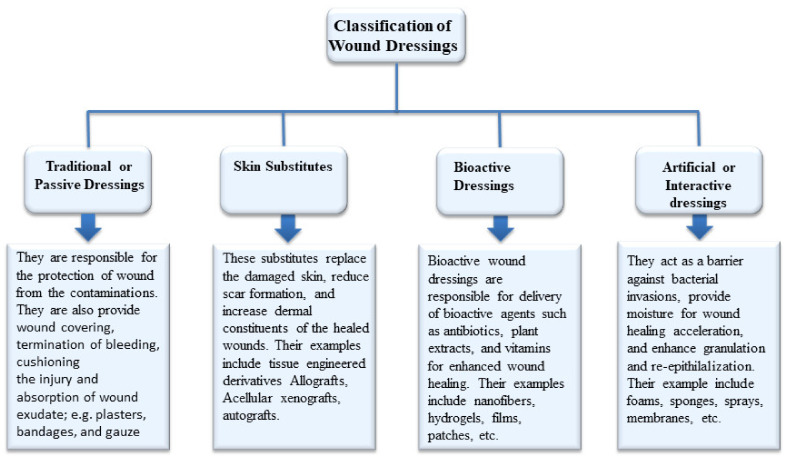
Classification of wound dressings.

Wound dressings act as barriers to protect the wound and also prevent contamination and infection. Due to the significance of wound protection on the accelerated wound healing process, the development of appropriate wound dressings is critical [25]. Potential wound dressings are classified based on various factors into four different types (Figure 2): traditional dressings (i.e., gauze and gauze–cotton composites), biomaterial-based dressings (i.e., acellular xenografts, allografts, autografts, and tissue derivatives), artificial dressings (i.e., foam, gel, membrane, film, and spray, composites), and bioactive dressings (i.e., hydrogels, transdermal patches, nanofibers, wafers) [26,27,28,29].

Traditional wound dressing products such as gauze, plasters, lint, synthetic/natural bandages, and cotton wool are dry and commonly used as primary/secondary dressings. Gauze dressings are characterized by high absorption capacity and made of woven or non-woven fibers of cotton, polyester, and rayon. Gauze dressings need to be frequently changed to avoid maceration of healthy tissues and cause repetitive tissue damage [30,31,32]. Biomaterial-based wound dressings are classified as xenografts, allografts, autografts, and tissue derivatives. Allografts are the skin tissues transplanted from one person to another, and their use is limited by the high risk of infections and disease transmissions [33]. Other significant clinical limitations include immune rejection, pain, scarring, and slow healing [34]. Tissue derivatives are originally derived from collagen and can cause infections after prolonged usage [32]. Artificial dressings, commonly known as interactive wound dressings, are prepared from synthetic polymers and biopolymers. The most commonly-used biopolymers include gelatin, chitosan, alginate, etc. Artificial dressings act as a barrier against microbial infections, improve the physiological environment of the wound, maintain a moist wound environment, enhance granulation and reepithelialization, and improve water vapor transmission rate with good tensile strength [35,36]. High risk of infections and lack of stability are the major disadvantages associated with traditional and biomaterial-based dressings. Developments of active wound dressings that can protect wounds from micro pathogens to overcome these drawbacks are needed. The bioactive wound dressings are dressing materials that deliver bioactive molecules to the targeted site. The bioactive agents that can be transported by these wound dressings include antibiotics, antioxidants, growth factors, stem cells, vitamins, and plant extracts, etc. [37]. Examples of bioactive dressings include hydrogels, nanofibers, membranes, films, transdermal patches, foams, and wafers. They are prepared from various polymers such as alginate, chitosan, elastin, dextran, hyaluronic acid, pectin, silk fibroin, and collagen. The properties of bioactive dressings include good biocompatibility, biocompatibility, patient compliance, and skin and environmental friendly [37,38]. The polymer-based bioactive wound dressings that are discussed in this review were loaded with AV plant extract for accelerated wound healing.

## 4. Biological Activities and Clinical Studies of *Aloe vera* in Wound Management

**Figure 3:**
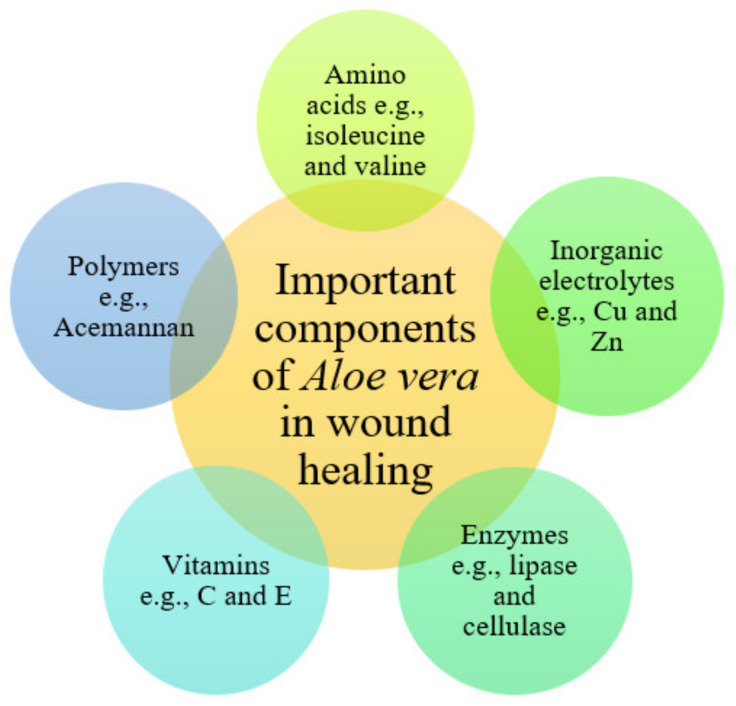
Essential Components of *Aloe vera.*

*Aloe vera* (L.) Burm. f. (Asphodelaceae) is a xerophytic plant commonly found in the arid regions of Africa, Europe, the Americas, and Asia. It is cultivated in India, Rajasthan, Andhra Pradesh, Gujarat, Maharashtra, and Tamil Nadu. In South Africa, AV has been used in traditional medicine for skin problems such as skin lesions and itching, and other ailments [39]. The biological activities of AV have been extensively studied, and most reports have revealed its multiple pharmacological activities such as anti-inflammatory, anti-diabetic, antibacterial, antioxidant, antiviral, and wound healing activities [40,41,42]. Wound healing activity is one significant prescription of AV gel use in various countries [43]. AV contains amino acids (e.g., glutamine threonine, isoleucine, valine, and phenylalanine), enzymes (e.g., bradykinase, catalase, lipase, cellulase, carboxypeptidase, and peroxidase), polymers (e.g., Acemannan and aloverose), and constituents vital for the wound healing process (Figure 3). It also has various inorganic electrolytes such as chromium, copper, potassium, iron, magnesium, zinc, sodium, and calcium, which are also essential in the wound healing process [44].

Various studies have revealed the accelerated wound healing process of AV [45,46,47,48,49,50,51,52,53,54]. AV prevents scar formation during skin injury by stimulating cell production and promoting the regeneration process at the deepest skin layers [46]. Various mechanisms of action have been reported for the wound healing activities of AV gel, such as increased epithelial cell migration, keeping the wound moist, reduction of inflammation, and rapid maturation of collagen [47]. Acemannan, one of the principal active constituents in AV gel, accelerates wound healing and activates macrophages to stimulate fibrotic cytokine production [48]. Moriyama et al. demonstrated AV beneficial effects on cell proliferation, development, differentiation, and wound healing on the epidermal keratinocytes [49]. Another active compound in AV gel, called mannose-6-phosphate, plays a significant role in the treatment of 1st and 2nd-degree burns [50]. A topical cream prepared from AV cured human 2nd-degree burn wounds with a healing rate of over one-half better than silver sulfadiazine [51]. The studies mentioned above clearly demonstrated the healing potential of AV and identified its active constituents responsible for wound healing. Furthermore, it has been shown that AV hinders thromboxane, which acts as a wound healing inhibitor and results in improved wound healing mechanisms. The constituent of AV called Magnesium lactate can stop the production of histamine that results in irritation and itching of the skin [52]. AV is also a potent inhibitor of inflammatory reactions by hindering IL-6 and IL-8 and decreasing TNF alpha levels [53]. AV is not only responsible for increasing the quantity of collagen in injuries but also changes the collagen composition, increases collagen cross-linking, and thereby leads to wound recovery [14]. In addition, polymers, together with zinc and amino acids, present in AV can result in moisture retention, skin integrity, reduction of erythema, and benefits in the prevention of skin ulcers [54].

Some clinical studies have shown the potential wound healing activity of AV. Eshghi et al. reported the wound healing activity of AV after surgery on 49 patients. The AV cream significantly reduced the pain after one- and two-days post-surgery. Furthermore, the Wound healing at the end of the second postoperative week was superior in the patients treated with AV cream compared to those treated placebo cream, demonstrating that AV possesses good wound healing activity [55]. Burusapat et al. examined the wound healing effects of AV gel and placebo on patients who undertook split-thickness skin graft harvesting. The clinical outcomes demonstrated that AV gel significantly accelerated split-thickness skin graft donor-site healing compared to the placebo group, although the formulation did not exhibit significant pain relief [56]. Molazem et al. conducted clinical studies on dressing containing AV on 90 women that underwent a cesarean operation. The AV-loaded dressing demonstrated effective cesarean wound healing compared to the pristine dressing. The topical application of AV gel did induce any side effects indicating its safety in wound healing [57].

The clinical study conducted by Khorasani et al. on 30 patients with burn wounds exhibited that 100% of the group treated with AV cream 0.5% were healed after 19 days while it was only 80% for sulfadiazine 1%. The mean days of healing for the AV and sulfadiazine groups were 15.9 ± 2 and 18.73 ± 2.56 days, respectively, without any infection noticed in both groups [58]. A similar study was conducted by Malek Hosseini et al. on 64 patients with 2nd-degree burns, and the outcomes showed that wounds treated with AV gel healed faster compared to those treated with silver sulfadiazine [59]. Rahmani et al. conducted prospective clinical trials to assess the effects of a topical cream containing 0.5% AV juice powder in treating chronic anal fissures. The results showed that patients’ pain in the AV cream group was dramatically relieved after one week of treatment, and wound healing time was significantly decreased compared to the non-aloe cream group [60]. Xu et al. investigated the clinical efficiency of AV on 2nd-degree burns. The results suggested that a combination of scald ointment and powder of AV internal layer showed higher therapeutic efficacy and provided faster recovery than treatment with the scald ointment alone [61].

## 5. Polymer-Based Wound Dressings Scaffolds Enriched with *Aloe vera*

### 5.1. Nanofibers/Nanofibrous Materials

Nanofibers are potential wound dressings that possess a mean fiber diameter that ranges from nanometer to few microns. They are considered ideal dressings due to their drug-delivery features [62,63]. These wound dressings offer physical protection to the injury site for an extended period and can be loaded with a high amount of bioactive agents for controlled drug release [63,64]. The other advantages of nanofiber wound dressings include high porosity, high surface-area-to-volume ratio, and small pore size [65]. Nanofibrous scaffolds mimic the extracellular matrix, thereby improving the proliferation of epithelial cells and new tissue development. Their nanometer fiber diameter stimulates hemostasis of damaged tissues, promotes dermal drug delivery, enhances fluid absorption, cell respiration, and high-gaseous exchange, thereby preventing bacterial invasion [66]. There are many reports on nanofiber materials loaded with AV for enhanced wound healing (Table 1).

Pathalamuthu et al. formulated chitosan-PEO hybrid nanofibers loaded with AV plant extract for wound care management [67]. The physicochemical properties of hybrid nanofibers were confirmed by FTIR and XRD analysis. The porosity analysis demonstrated a more uniform pore size in chitosan-PEO nanofibers with a pore size diameter of approximately 0.95 µm (97%) suitable for the absorption of wound exudates, gaseous exchange for cell proliferation, and re-epithelization. The wettability studies of the hybrid nanofibers demonstrated water contact angles less than 50°, indicating hydrophilicity that can result in high water uptake. The in vitro antibacterial experiments of AV-enriched nanofibers showed superior antimicrobial effects against *Staphylococcus aureus* (*S. aureus*) and *Escherichia coli* (*E. coli*). The in vivo wound-healing experiments using full-thickness wound in Swiss Albino mice demonstrated complete wound closure on day 15 when dressed with AV extract-loaded nanofibers while wound treated with control completely closed on day 25 [67].

Kheradvar et al. formulated silk fibroin-PVA hybrid nanofibers encapsulated with AV extract-loaded starch nanoparticles [68]. The SEM micrographs of the hybrid nanofibers showed continuous and beadless morphology (mimicking extracellular matrix) with a mean diameter of about 342.24 ± 5.27 nm. The in vitro drug release studies at physiological conditions (pH 7.5 and temperature 37 °C) demonstrated an initial burst release of AV from the hybrid nanofiber followed by a slow and sustained release. The in vitro antioxidant studies using DPPH radical scavenging showed that increasing the AV-loaded starch nanoparticles content significantly resulted in high antioxidant efficacy, which could have a positive effect on the wound-healing process [68]. Uslu et al. formulated PVA/PVP/PEG hybrid nanofibers loaded with AV gel for wound management. The SEM images of hybrid nanofibers demonstrated uniform beadless morphology with fiber diameters that range between 200–500 nm. These results suggested that the beadless morphology can lead to increased porosity and promote oxygen permeation and moisture to the injury, vital factors for the wound healing process [69].

Serincay et al. developed PVA-PAA hybrid electrospun nanofibers encapsulated with AV gel and ciprofloxacin for wound dressing application. The drug release studies in vitro displayed a controlled release of ciprofloxacin ad AV gel from the nanofibers. The in vitro antimicrobial analysis of dual-drug loaded nanofibers using the disc diffusion method demonstrated excellent antibacterial effects against *Psudonomas aeruginosa (P. aeruginosa)*, *S. aureus*, and *E. coli*, suggesting that these nanofibers are suitable antibacterial wound dressing materials [70]. The combination of AV gel and ciprofloxacin demonstrated superior antibacterial activity of the wound dressing nanofibers due to their synergistic effect. Despite this, the amount of AV gel must be considered because its high quantity in the dressing can decrease the drug release of ciprofloxacin, leading to poor antibacterial activity [70]. Baghersad et al. fabricated gelatin-PCL electrospun hybrid nanofibers loaded with AV gel. The in vitro cell proliferation analysis demonstrated high cell viability and proliferation of NIH-3T3 fibroblasts when incubated with AV-loaded hybrid nanofibers, showing that AV significantly improves the biocompatibility of the nanofibers. The antibacterial studies in vitro using the Broth dilution test demonstrated that AV-enriched hybrid nanofibers had more than 99% and 85% antibacterial efficacy against *S. aureus* and *E. coli,* respectively [71]. Nikbakht and co-workers formulated chitosan-PEO nanofibers encapsulated with AV for wound management. The in vitro drug release studies at physiological conditions demonstrated an initial rapid release of AV from the hybrid nanofibers followed by sustained release. The in vitro cytocompatibility experiments exhibited high cell viability and proliferation of L929 murine fibroblasts when immersed with AV-enriched hybrid nanofibers, suggesting excellent biocompatibility and non-toxicity [72].

Rafieian et al. reported chitosan-PVA nanofibers containing AV extract for wound dressing application. The in vitro antimicrobial analysis of AV extract-loaded nanofibers demonstrated excellent bactericidal effects against *E. coli* and *S. aureus*. The swelling analysis of the nanofibers displayed an increased swelling ratio with high water uptake capacity, which is probably caused by the hydrophilic nature of AV extract. The mechanical characterization analysis of AV extract-loaded nanofibers demonstrated tensile strength of 49.81 ± 1.09 MPa, Young’s modulus of 22.49 ± 2.16, and elongation at break of 4.33 ± 0.53%, revealing that these nanofibers are suitable for the human skin and can be easily handled during wound dressing application [73]. Ranjbar-Mohammadi et al. designed PCL-gum tragacanth electrospun nanofibers loaded with AV extract. The SEM analysis showed that increasing AV extract content in the electrospun hybrid nanofibers decreased the average fiber diameter from 184 ± 34 to 123 ± 22 nm. The FTIR and differential scanning calorimetry (DSC) confirmed the successful loading of AV in the hybrid nanofibers. The in vitro cytotoxicity assay demonstrated high cell proliferation and viability of NIH 3T3 fibroblast cells when incubated with AV-loaded nanofibers compared to the unloaded nanofibers [74]. The AV extract-loaded PVA nanofibers reported by Sirima et al. demonstrated excellent wound healing properties (such as swelling capacity and drug release profile) compared to AV-loaded PVA hydrogels [75]. The electrospun fibers displayed smooth surface, non-woven, and high surface area. The rate of AV release from the electrospun fibers was rapid compared to the hydrogel films in vitro. The finding mentioned above is attributed to the large surface area of the electrospun fibers [75].

Bootdee and Nithitanakul developed AV extract-enriched PVA nanofibers loaded PLGA nanocrystals for wound dressing. The in vitro drug release profile demonstrated a prolonged drug release profile with the addition of PLGA nanospheres. The in vitro antimicrobial studies demonstrated good antibacterial effects against *S. aureus* and *E. coli* [76]. Ezhilarasu et al. synthesized AV extract-enriched PCL nanofibers loaded with curcumin and tetracycline hydrochloride for wound management. FTIR spectra of electrospun nanofibers confirmed the successful encapsulation of AV, curcumin, and tetracycline hydrochloride into the PCL nanofibers. The SEM images showed bead-free morphology with an average fiber diameter size of about 770 nm. The in vitro studies demonstrated high antibacterial efficacy against *E. coli* and *S. aureus* with increased cell proliferation and viability of human dermal fibroblasts when immersed in AV-enriched PCL nanofibers loaded with curcumin and tetracycline hydrochloride [77]. The loading of tetracycline in nanofibers together with AV extract significantly improved the antibacterial activity. Ghorban et al. prepared Zein/PCL/Collagen electrospun hybrid nanofibers encapsulated with AV extract and zinc oxide nanoparticles for wound management. The mechanical properties analysis demonstrated tensile strength that ranged between 1.21 MPa and 3.94 MPa, which increased with an increase in the content of PCL. The antibacterial studies showed that the dual drug-loaded nanofibers revealed high inhibition zones against *S. aureus* and *E. coli* bacterial strains compared to the plain nanofiber which did not show any significant inhibition zone [78].

Yin and Xu fabricated electrospun PCL-chitosan nanofiber membranes incorporated with AV extract for wound healing application [79]. The contact angle measurements demonstrated that the presence of AV and chitosan significantly enhance the hydrophilic nature of nanofiber membranes, which could stimulate the attachments and growth of skin cells. The porosity analysis showed that AV-loaded nanofibrous membranes had a larger mean flow pore diameter than the plain nanofibrous membranes, leading to high porosity and moderate water vapor transmission rate (WVTR). The in vitro antibacterial experiments demonstrated the inhibition rates of plain nanofiber membranes and AV extract-loaded nanofibrous membranes against *E. coli* were 92.92% and 96.68%, respectively, indicating that they can be potential wound dressings for the treatment of bacterial-infected wounds [79]. Garcia-Orue et al. formulated AV extract-enriched PLGA nanofiber membranes loaded with lipid nanoparticles. The in vitro cytotoxicity studies using MTT assay demonstrated more than 70% cell viability of keratinocytes and fibroblasts when incubated with nanofiber membranes, suggesting good biocompatibility. The in vivo wound healing studies in full-thickness wound mice model revealed that wounds dressed with the nanofiber membranes were almost closed on day 15 (wound closure was 95.53 ± 4.96% for the wounds treated with AV-enriched nanofiber membranes and 91.95 ± 6.08% for *Aloe vera*-enriched nanofiber membranes loaded with lipid nanoparticles, respectively). In comparison, the untreated wounds demonstrated only closure of 62.19 ± 11.84% [80]. Furthermore, Garcia-Orue et al. prepared PLGA nanofibrous membranes co-loaded with recombinant human Epidermal Growth Factor and AV extract for wound management. The in vivo studies of the dual bioactive agent-loaded nanofiber membranes demonstrated significantly accelerated wound closure and reepithelization in full-thickness wound healing performed in vivo [81]. The encapsulation of recombinant human Epidermal Growth Factor in AV-loaded nanofibers significantly resulted in fast wound reepithelization, which is very important in wound healing.

Naseri-Nosar et al. prepared chitosan-PVA electrospun nanofiber sponge-like wound dressings loaded with AV gel and erythropoietin. These nanofibrous scaffolds showed WVTR and water-uptake capacity of 584.00 ± 144.67 gm^−2^ and 26.40% ± 3.37%, respectively. The mechanical properties of the nanofiber sponge-like wound dressings revealed a tensile strength of 1.69 ± 0.04 MPa that is suitable for skin. The in vivo wound closure studies showed that the hybrid nanofibrous dressings loaded with AV gel and erythropoietin had a significantly higher wound closure of about 92.96% ± 10.09% compared to the sterile gauze with 73.89% ± 2.61% wound closure after two weeks [82]. The combination of AV gel and erythropoietin in one wound dressing significantly caused the acceleration of wound recovery in vivo. Isfahani et al. formulated PVA electrospun nanofibrous pads loaded with AV extract for control release in wound treatment. The FTIR and XRD data confirmed the successful formulation of the nanofibrous pads. The SEM micrographs of AV-loaded pads showed bead-free morphology with an average fiber diameter of 110 nm. The in vitro drug release profile showed that 60% of AV was released from the nanofibrous pads within an hour, and 90% was released between 2–4 h, suggesting that these AV-enriched PVA nanofibrous pads are potential materials for wound dressing application [83].

There are many interesting outcomes of AV-loaded nanofibers that are demonstrated by the studies reported by several researchers. The hydrophilic nature of AV extract significantly resulted in increased swelling and high water uptake capacity of nanofibers that make them suitable for the treatment of wounds with excess exudates. In addition, the hydrophilic nature of AV also improved the hydrophilicity of nanofibers, promoting skin cell proliferation and adhesion. The encapsulation of AV in nanofibers decreased the fiber diameter, suitable for wound treatment because it bio-mimic extracellular matrix. The cytocompatibility studies have shown that the AV-loaded nanofibers are non-toxic to skin cells, demonstrating their safety in wound skin wound care. The antibacterial activities of nanofibers loaded AV was high compared with plain nanofibers. The combination of AV extracts with other drugs resulted in a synergistic antibacterial effect. Furthermore, the antibacterial activity of the nanofibers loaded with AV was influenced by the rate of drug release from the nanofibers.

### 5.2. Films/Membranes

Films are wound dressings that are fabricated from transparent and adherent PU, which provides a suitable gaseous exchange between wound and environment [84]. The transparency of film dressing allows the monitoring of the wound healing process without its removal. The advantages of film wound dressings include their excellent mechanical properties, i.e., high flexibility and elasticity, resulting in their ability to be changed to any shape with no additional tapping requirement. However, these dressings are not appropriate for high exudate wounds because they cannot absorb high amounts of biological fluids due to their low porosity [4]. Some researchers reported the efficacy of films loaded with AV in wound management (Table 1).

Liu et al. formulated chitosan-AV extract films loaded with curcumin-encapsulated microspheres for wound healing and skin regeneration [85]. The XRD and FTIR data confirmed the successful formulation of the films. The SEM images of the films exhibited a smooth, continuous, and uniform morphology, and the microspheres were dispersed uniformly on the surface of the films, indicating their successful encapsulation. The porosity of the films ranged between 29.52% and 43.47%. The in vitro antibacterial activity of the films against *E. coli* and *S aureus* was significantly improved by the incorporation of curcumin-loaded microspheres. The in vitro cell proliferation studies using CCK-8 assay showed high cell proliferation with cell viability of about 112.49% for NIH-3T3 cells when incubated with films, suggesting excellent biocompatibility. The full-thickness skin wounds in the rat model treated with films demonstrated better and faster recovery than those dressed with gauze (control) group after seven days [85]. The co-encapsulation of curcumin-loaded microspheres with AV extract significantly improved the antibacterial efficacy of films with a fast wound healing process.

Hajian et al. developed PVA-based hydrogel films incorporated with AV extract as potential wound dressings. The developed films displayed high transparency in both wet and dry states with suitable flexibility, indicating good mechanical performance. The transparency of the films allowed the injury to be easily monitored without removing the dressing. The increase in AV content up to 30% significantly increased the water absorption of the films resulting from the hydrophilic properties of AV. The increased AV content improved the mechanical properties of films. The in vitro degradation studies of the films revealed a rapid weight loss on the first day. The in vitro MTT assay demonstrated that the encapsulation of AV in the films significantly promoted the cell proliferation of fibroblasts, and this could stimulate rapid wound healing [86]. Pereira et al. formulated alginate films incorporated with AV gel using the casting solvent method. The thickness of the films ranged between 29.00 ± 2.80 µm and 39.33 ± 4.58 µm. The SEM micrographs revealed the surface morphology of the plain alginate films, which was uniform and smooth, while the AV-incorporated films displayed a rough surface because of the presence of AV. The tensile strength values of the films were in a range of 48.04 ± 3.15 MPa and 59.46 ± 1.12 MPa, suggesting good mechanical performance that is suitable for wound management. The water uptake analysis showed that the AV significantly contributes to an increase in the film water absorption capacity, indicating their potential to absorb wound exudates [87]. Khoshgozaran-Abras et al. prepared chitosan-based films incorporated with different contents of AV extract. The thickness of the films was affected by AV incorporation. An increase in the ratio of AV versus chitosan from 0% to 50% resulted in a significant reduction in the thickness of the films from 0.213 ± 0.003 mm for pristine chitosan film to 0.163 ± 0.005 mm for film incorporated with 50% AV. The water vapor transmission analysis showed that the increase in the content of AV slightly reduces the WVTR of films that can lead to suitable moisture for accelerated wound healing. The increase in the amount of AV up to 20% significantly improved the mechanical properties of films with tensile strength, elastic modulus, and elongation at a break of 8.49 ± 0.70 MPa, 10.50 ± 0.42 MPa, and 80.89 ± 7.72%, respectively [88].

Pereira et al. designed alginate–PVA hybrid films incorporated with AV extract and vitamin E for wound treatment. The in vitro drug release profile was a rapid release of vitamin E from the hybrid films followed by sustained release. The film dressings promoted a deeper accumulation of vitamin E in the stratum corneum when compared with a traditional semisolid formulation, indicating that these films are capable of delivering bioactive agents to stimulate the wound healing process [89]. Thomas et al. developed alginate-based films loaded with AV gel and cellulose nanocrystals for wound healing application. These polymeric films exhibited a tensile strength of 5.38 MPa and elongation at a break of about 7.95%, indicating suitable mechanical properties for wound dressing application. The in vitro studies of the dual drug-loaded films demonstrated superior antibacterial efficacy against *S. aureus* than *E. coli* with high cell viability of L929 fibroblasts indicating good biocompatibility. The scratch wound assay showed that the RAW-264.7 cells incubated with films proliferated significantly quicker than those immersed in the control group, suggesting a rapid wound healing process [90]. The chitosan–alginate films co-encapsulated with AV and Ag nanoparticles developed by Chabala et al. demonstrated excellent antibacterial activity against *S. aureus* and *P. auregonosa*, indicating that these dressings can be used to treat bacteria-infected wounds [91]. The combination of AV and nanoparticles can result in synergistic antibacterial effects against several bacterial strains that usually invade chronic wounds.

Pereira et al. formulated alginate hydrogel films encapsulated with AV gel, and these films displayed a smooth and homogeneous surface. The FTIR data confirmed the successful formulation of AV gel-loaded films. The water uptake studies exhibited significantly higher water absorption for polymeric films containing high AV contents (15% and 25%), indicating their potential to absorb wound exudates [92]. The alginate-based hydrogel films loaded with AV gel formulated by Pereira demonstrated elongation at a break that ranged between 7.86 and 13.56%, and tensile strength ranging between 5.70 MPa and 6.58 MPa that is in the range of human skin mechanical properties. The contact angle values of films were between 33.88° and 50.91°, suggesting the hydrophilic properties that can be beneficial for high water uptake. Furthermore, the results from this study demonstrated that the increase in the AV gel content in films significantly enhanced the film transparency that is good for wound observation without removing film dressings [93].

Gupta et al. reported PVA-PEO-carboxymethyl cellulose-polyester non-woven hybrid membranes loaded with AV extract. The SEM micrograph revealed a three-dimensional irregular interconnected porous morphology. The water uptake studies of the membranes demonstrated high swelling capacity suitable for wound exudate absorption and provision of moisture at the wound bed to accelerate wound healing. The in vitro antimicrobial studies of AV-loaded hybrid membranes showed a high zone of inhibition against *S. aureus* and *E. coli*, indicating that these membranes can be suitable for bacterial-infected wound treatment [94]. Gupta et al. also reported that the drug release of curcumin from AV extract-enriched PVA-PEO-carboxymethyl cellulose-polyester non-woven hybrid membranes was an initial burst release for 24 h followed by a sustained drug release mechanism. These dressings displayed WVTR that ranged between 2000 and 2500 gm^−2^ day^−1,^ which is suitable for a moist environment for the acceleration of wound healing [95].

Ranjbar and Yousefi prepared chitosan nanoparticle thin-film membranes enriched with AV gel for wound healing applications. The in vivo studies using the rat model demonstrated that the MRSA-infected full-thickness wounds healed significantly faster when treated with AV-loaded membranes than those dressed with saline [96]. Singh and co-workers prepared dextran-based bionanocomposite membranes encapsulated with AV extract and Manuka honey for wound treatment. The in vitro drug release profile demonstrated initial burst release behavior for both bioactive from the membranes followed by controlled release. These dual drug-loaded membrane dressings displayed more than 99% bactericidal effect against *E. coli* and *S. aureus,* while plain membranes showed only 73% bactericidal effect. The WVTR value of the membrane was 3820 ± 69.64 g/m^2^/day, which is suitable for wound healing. The in vivo wound healing experiments using full-thickness wounds in mice model showed that AV-loaded membranes significantly improved epithelialization by scar prevention and aesthetics with complete wound closure after the 14th post-surgery day [97].

The cytotoxicity studies of AV-loaded polymeric films/membranes demonstrated very high cell viability, indicating that these scaffolds are non-toxic and safe when loaded with AV. The incorporation of AV in the films did not affect their transparency and enhanced their mechanical performance that is suitable for wound management. In some studies, the increase in the amount of AV significantly increased the water absorption capacity of the films. An increase in AV content slightly reduced the WVTR of films/membranes, resulting in dressings that provide suitable moisture for accelerated wound healing. In addition, the AV-loaded films/membranes exhibited excellent antibacterial and wound healing effects, especially in the case of combination therapy in wound dressings.

### 5.3. Hydrogels

Hydrogels are hydrophilic three-dimensional networks with an excellent capacity to absorb and retain large amounts of biological fluids and water without dissolving [98]. They can be tailored to be chemically stable or biodegrade in the presence of biological fluids over some time. Several advantages are displayed by hydrogels in the field of wound management, including high porosity, the high adsorption capacity of wound exudate, high storage capacity, acts as a barrier against microorganisms, biocompatibility and biodegradability, specific environmental stimuli-responsiveness, for example, pH, temperature, etc. to stimulate drug release continuously into the infected wound site [99,100]. Despite the unique properties of hydrogels that make them useful in wound dressing, they also suffer from some shortcomings, such as poor mechanical stability at swollen state, ability to dehydrate if not covered, suggesting the need for a secondary dressing, and their ability to cause skin maceration, making them difficult to secure [101]. To overcome the poor mechanical stability of hydrogels, they are prepared by a combination of synthetic and natural polymers resulting in hydrogels with good mechanical properties. There are some polymer-based hydrogels loaded with aloe vera for wound treatment (Table 1).

Anjum et al. fabricated polymethacrylic acid-based nanosilver nanohydrogels loaded with AV gel and curcumin using nanoemulsion polymerization for wound healing application [102]. The FTIR data confirmed the successful formulation of nanohydrogels. The in vitro drug release studies demonstrated that the release of the silver ions was via initial burst release from the hydrogels followed by sustained release. The in vitro antimicrobial studies using the colony count method demonstrated 100% antibacterial efficacy against *S. aureus* and more than 98% against *E. coli*, indicating synergistic antibacterial effects of the loaded bioactive agents and nanosilver in the hydrogels. The in vivo studies using albino Swiss mice showed that the wounds treated with dual drug-loaded nanosilver nanohydrogels were 100% closed on day 16. In comparison, those treated with plain nanosilver nanohydrogels were closed by only 75%. The histology analysis of the dual drug-loaded nanosilver nanohydrogels demonstrated that the treated wound tissues showed a thinner epidermal layer and organized collagen deposition with the absence of inflammation [102]. The loading of more bioactive agents together with AV gel resulted in synergetic antibacterial effects against the bacterial strains that are common in wounds and eventually lead to a fast wound healing process.

Dadashzadeh et al. fabricated alginate-gelatin hybrid hydrogels incorporated with AV extract-loaded niosomes for skin wound treatment [95]. The Dynamic Light Scattering characterizations of AV extract-loaded niosomes displayed polydispersity index, size, and encapsulation efficiency of 0.108, 270.080 nm, and 2.039 ± 4.090%, respectively. The swelling analysis demonstrated that the water absorption rate of the hydrogels incorporated with AV-loaded niosomes displayed an increased water uptake for 3 days, indicating that these hydrogels can absorb excess wound exudates. The in vitro biodegradation studies showed that the hydrogel degradation rate was gradual. The mechanical analysis of hydrogels showed a mean Young modulus of approximately 12.64 ± 1.3 kPa, in the range of human skin (7–33 kPa). The in vitro cytotoxicity studies using MTT assay showed high cell viability and proliferation of fibroblast cells when incubated with hydrogels incorporated with AV-loaded niosomes. These hydrogels are potential wound dressings with excellent biocompatibility [103].

Dey et al. developed poly(*N*-vinylpyrrolidone-acrylamide) copolymer hydrogels loaded with AV gel. The swelling study of hydrogels showed that the swelling rates decreased with the increasing content of AV in the polymeric matrix. The SEM analysis of hydrogels displayed swollen morphology with interconnected pores that can promote gaseous exchange, cell migration, and proliferation during wound healing [104]. Bialik-Wąs et al. prepared sodium alginate-PVA hybrid hydrogels loaded with AV. The AV-loaded hydrogels demonstrated high fluid uptake and swelling capacity. The mechanical characterization analysis showed that the hydrogels possessed good tensile strength and elongation at a break that is suitable for wound management. The in vitro drug release studies showed an initial rapid release of AV components from the hydrogels followed by slow release. The cell viability of human fibroblasts in the presence of AV-loaded hydrogels was between 80–90%, suggesting excellent biocompatibility and non-toxicity. The results of this study revealed the outstanding characteristics of an ideal wound dressing loaded with AV [105]. The AV-encapsulated polymer-based hydrogels displayed excellent antimicrobial properties that are useful in the treatment of infected wounds. The AV content significantly improved the water uptake of the hydrogels, making them suitable for the treatment of wounds with excess exudate. In some cases, the increased AV content decreased the swelling rate.

### 5.4. Others

There are also various types of dressings loaded with AV for wound treatment, including sponges, bandages, foams, wafers, patches, etc. Anbazhagan and Thangavelu formulated AV extract-enriched chitosan composite sponges loaded with tetracycline hydrochloride for wound management [106]. The successful formulation of composite sponges was confirmed by FTIR. The SEM micrographs of AV-enriched sponges displayed well-interconnected porous morphology. The porosity analysis showed a porosity of about 68.74%, beneficial for gaseous exchange, wound exudate, cell proliferation, and migration. The in vitro drug release profile demonstrated that tetracycline hydrochloride release was gradually increased from the AV-enriched sponges. The AV-enriched sponges loaded with tetracycline hydrochloride showed higher inhibitory action against *E. coli*, *S. aureus*, *K. pneumoniae*, and *B. subtilis* compared with the plain chitosan sponges indicating their potential application as antibacterial wound dressings [106]. The loading of antibiotics, tetracycline, significantly improved the antimicrobial activity of AV-loaded sponges because of the combination therapy of antibiotic and AV extract. Salah et al. developed cellulose-based cotton gauze grafted with AV acemannan extract for wound management. The successful grafting of AV into gauze was confirmed by the FTIR. The cotton gauze did not display toxic effects when incubated with HepG2 cells using MTT assay. The antimicrobial studies of AV-grafted gauze exhibited a high inhibitory effect of 70.2% and 72.4% against *S. aureus* and *E. coli*, respectively [107].

Tummalapalli et al. reported pectin-gelatin hybrid biocomposite dressings co-loaded with AV extract and curcumin for wound treatment. The antioxidant analysis of biocomposites demonstrated about 80% free radical scavenging efficacy with good antibacterial effects against *S. aureus* and *E. coli*. The biocomposite demonstrated high cell viability when incubated with NIH3T3 mouse fibroblasts, suggesting good cytocompatibility. The in vivo studies using the mice model showed that the co-loaded biocomposites significantly accelerated wound healing of full-thickness wounds with about 80% wound closure on day 8 post wounding [108]. The co-encapsulation of AV extract and curcumin significantly resulted in good biological activities (antioxidant and antibacterial efficacy) that can be beneficial in wound treatment, especial for chronic wounds. Ghayempour et al. reported tragacanth gum nanocapsules encapsulated with AV. The SEM micrograph pictures confirmed the successful preparation of the nanocapsules. The encapsulation efficiency of 91% suggested that AV was successfully encapsulated in tragacanth gum nanocapsules. The wound-healing studies of AV-encapsulated nanocapsules using scratch assay showed a high migration rate of fibroblasts to the scratch, indicating a rapid wound healing process [109]. Ghayempour et al. developed cotton fabric dressings loaded with AV extract-encapsulated tragacanth gum nanocapsules for wound treatment. The SEM micrographs revealed the successful development of AV-loaded nanocapsules on the cotton fabric dressings with spherical shape and mean size ranging between 55 and 70 nm. The in vitro scratch assay demonstrated excellent wound healing effects of the cotton fabric dressings loaded with AV-encapsulated nanocapsules with a cell migration rate of 88% after 24 h [110].

Abdel-Mohsen et al. developed chitosan-glucan complex hollow fibers reinforced collagen wound dressings encapsulated with AV extract. These dressings demonstrated rapid swelling rate, and higher water uptake, indicating their potential application for the management of high exuding wounds [111]. Rubio-Elizalde et al. designed alginate-PEG methyl ether methacrylate biocomposite wound dressings, enriched with AV gel and *Moringa oleifera* extract. The in vitro cytotoxicity studies using MTT assay showed high cell viability of CCD-1112Sk human skin fibroblasts when incubated for 10 days with dual drug-loaded biocomposites compared with the plain biocomposite, suggesting non-toxicity and good biocompatibility of the biocomposite dressings as ideal wound dressings. The in vitro antimicrobial experiments of the biocomposite wound dressings loaded with AV and *Moringa oleifera* displayed good antibacterial activity against *E. coli* and *S. aureus.* In contrast, the plain biocomposites did not reveal any significant antibacterial effects [112]. The dual loading of AV gel and *Moringa oleifera* resulted in good antibacterial activity compared with pristine biocomposite. The other polymer-based wound dressings such as sponges, hallow fiber, and biocomposites loaded with AV extracts, also displayed good wound healing properties. These dressing demonstrated excellent biocompatibility that makes them suitable ideal wound dressings. The loading of AV also enhances the biological activities (antibacterial and antioxidant efficacy) of the dressings with improved wound healing effects.

**Table 1:** Summary of AV-enriched polymer-based wound dressings.

Types of Wound Dressings Loaded with AV	Polymers Used	Effectiveness/Efficacy of Dressing	Harmfulness/Safety of Dressing	Ref
Nanofibers	Chitosan and PEO	Superior antibacterial efficacy against *S. aureus* and *E. coli* with fast full-thickness wound healing process.	The histological studies demonstrated high cell proliferation and increase blood vessels, indicating non-toxicity.	[67]
	Silk fibroin and PVA	High antioxidant activity that can result in reduced toxic oxidation products in chronic wounds	These nanofibers were harmless when were incubated with fibroblasts, suggesting their safety in wound care.	[68]
	PVA, PVP, and PEG	No biological activities reported, but porosity was high and can promote acceleration of wound by stimulating high gaseous exchange and wound exudate absorption.	No cytotoxicity experiments reported.	[69]
	PVA and PAA	They were very effective against microbial strains *(P. aeruginosa S. aureus*, and *E. coli*))	The cytotoxicity studies were not reported	[70]
	Gelatin and PCL	They were very effective against *S. aureus* and *E. coli* bacterial strains.	These scaffolds are safe because they showed high cell viability of fibroblasts.	[71]
	Chitosan and PEO	The initial burst drug release of AV can result in good biological efficacies.	The biocompatibility studies demonstrated non-toxicity on murine fibroblast cells.	[72]
	Chitosan and PVA	Excellent antibacterial efficacy against *S. aureus* and *E. coli*.	The nanofibers are safe to be used in wound healing due to their non-toxicity on murine fibroblasts.	[73]
	Gum tragacanth and PVA	These nanofibers can be effective in wound healing application due to their ability to absorb exudate	There was high cell proliferation of skin cells indicating good biocompatibility	[74]
	PVA	The fast release of AV can lead to good biological activities.	Not available	[75]
	PVA	Good antibacterial effectiveness against *S. aureus* and *E. coli.*	Not reported	[76]
	PCL	Excellent antibacterial efficacy against *E. coli* and *S. aureus.*	High cell proliferation and viability of human dermal fibroblasts indicating safety in the field of wound healing.	[77]
	Zein, PCL, and Collagen	High inhibition zones against *S. aureus* and *E. coli,* suggesting excellent antibacterial efficacy.	Cell adhesion and proliferation studies displayed no toxicity effect on fibroblasts, indicating that these nanofibers are harmless.	[78]
Nanofiber membranes	Chitosan and PCL	Excellent bactericidal efficacy against *E. coli*.	Nanofibers were harmless on human umbilical vein endothelial cells, demonstrating their safety.	[79]
	PLGA	Acceleration of full-thickness wound healing process.	The nanofibers were non-toxic although they showed a slightly low cell viability of 70%.	[80]
	PLGA	Fast wound recovery and reepithelization in full-thickness wound healing	Cell adhesion studies showed a high attachment of fibroblasts on nanofibers, showing non-toxicity.	[81]
Nanofiber sponge	Chitosan and PVA	higher wound healing mechanism.	Cytocompatibility studies toward skin cells showed non-toxicity of nanofibers making them suitable for wound-healing applications.	[82]
Nanofiber pads	PVA	Drug release studies demonstrated that these pads could result in good biological activities.	Not available	[83]
Films	Chitosan	Good antibacterial synergistic activity against *E. coli* and *S aureus* with fast wound recovery.	High cell viability of about 112.49% of fibroblast cells, indicating that these films are very safe.	[85]
	PVA	These films demonstrated favorable WVTR that can promote fast wound healing activity.	Good cell proliferation of fibroblasts showing non-toxicity.	[86]
	Alginate	High water uptake capacity that can reduce excess exudate to accelerate wound healing.	Not reported	[87]
	Chitosan	Appropriate WVTR that can lead to a fast wound-healing process	Not reported	[88]
	Alginate and PVA	Fast wound healing process	Not reported	[89]
	Alginate	Superior antibacterial efficacy against *S. aureus* than *E. coli.* Quick wound-healing process.	Excellent biocompatibility, indicating their safety.	[90]
	Chitosan and alginate	Excellent antibacterial activity against *S. aureus* and *P. auregonosa*	Good cytocompatibility, indicating non-toxicity.	[91]
	Alginate	Accelerated wound healing mechanism.	Not reported	[92]
	Alginate	faster wound healing mechanism.	Not reported	[93]
Membranes	PVA, PEO, and carboxymethyl cellulose	High antibacterial activity against *S. aureus* and *E. coli*.	The drug release studies showed that these scaffolds are non-toxic.	[94]
	PVA, PEO, and carboxymethyl cellulose	Moderate WVTR demonstrated that these dressings can promote fast wound healing.	The drug release profile displayed that these scaffolds are non-toxic.	[95]
	Chitosan	Quick MRSA-infected full-thickness wound healing process.	Histological studies demonstrated that these membranes are not harmful to skin cells.	[96]
	Dextran	Almost 100% bactericidal efficacy against both *E. coli* and *S. aureus,* with fast wound healing.	Good biocompatibility, showing safety to be used in wound treatment.	[97]
Hydrogels	Polymethacrylic acid	High antimicrobial efficacy of 100% against *S. aureus* and more than 98% against *E. coli,* and good wound healing effects.	The histopathological experiment showed that these wound dressing are non-toxic to skin cells.	[102]
	Alginate and gelatin	The quick biodegradation of these hydrogels can result in fast skin regeneration.	High cell viability and proliferation of fibroblast cells, indicating non-toxicity.	[103]
	poly (*N*-vinylpyrrolidone-Acrylamide) copolymer	Ability to induce wound healing.	Non-toxic.	[104]
	Alginate and PVA	Drug release profile demonstrated that these hydrogels could result in good biological activities.	Excellent biocompatibility and non-toxicity, indicating their safety	[105]
Composite sponges	Chitosan	Higher inhibitory action against *E. coli*, *S. aureus*, *K. pneumoniae*, and *B. subtilis.*	Good cytocompatibility, confirming that they are harmless.	[106]
Cotton gauze	Cellulose	Good antibacterial activity against *S. aureus* and *E. coli*.	Non-toxicity effects when incubated with HepG2 cells.	[107]
Biocomposite dressing	Pectin and gelatin	Good radical scavenging and antibacterial efficacy with accelerated wound healing.	High cell viability when incubated with fibroblasts, indicating harmlessness.	[108]
Nanocapsules	Tragacanth gum	Rapid wound healing activity.	High cell viability of human fibroblasts, indicating non-toxicity.	[109]
Cotton fabric dressings	Tragacanth gum	Good antimicrobial efficacy against *E. coli*, *S. aureus* and *C. albicans*.	Good biocompatibility that can demonstrate safety in wound treatment.	[110]
Hollow fibers	Collagen	Excellent wound healing efficacy.	Cell migration rate, demonstrating non-toxicity.	[111]
Biocomposite wound dressing	Alginate and PEG	Good antibacterial activity against *E. coli* and *S. aureus*	High cell viability of human skin fibroblasts, suggesting safety.	[112]

## 6. Conclusions

The wound dressings prepared from synthetic and natural polymers demonstrate interesting features such as high porosity, high fluid uptake, and high swelling ability that can promote migration and proliferation of skin cells and accelerate wound healing. The moderate WVTR of polymer-based dressings significantly prevents the accumulation of wound exudate that can result in bacterial invasion and prevent wound dehydration. These dressings usually suffer from poor biological activities that hamper their application in wound treatment. The biological properties (such as antibacterial, anti-inflammatory, and antioxidant efficacy) of polymer-based dressings can be improved by incorporating plant extract. The polymer-based dressings enriched with AV plant extract demonstrate excellent biological activities, such as antibacterial effects against various bacterial strains that normally infect wounds. However, the content of AV extract in polymer-based dressings must be considered because it can have some effects on mechanical performance and porosity. The in vitro studies have displayed very high cell viability of skin cells in the presence of AV-loaded polymer-based wound dressings, suggesting that these scaffolds are safe to be used in wound treatment. The loading of AV cannot affect the features of wound dressings, such as the transparency of films. The current reports on clinical studies of AV extract have shown its shortcoming is the inability to reduce pain during wound healing. Some in vitro series have demonstrated that AV can slow the drug release of the co-loaded drug, and this can result in a poor biological activity of the co-loaded bioactive agent. These limitations must be considered by biomedical researchers to make these materials suitable for clinical use. There are very few AV polymeric wound dressings that are in clinical applications, and the reported in vitro and in vivo studies demonstrate promising results. The combination of AV extract/gel with polymers can result in effective wound healing effects, making it beneficial for the design of wound dressings.
